# Supplementary material for: High‐Speed and Continuous‐Wave Programmable Luminescent Tags Based on Exclusive Room Temperature Phosphorescence (RTP)
Source: Adv Sci (Weinh). 2021 Oct 27;8(23):2102104. doi: 10.1002/advs.202102104 (PMC8655189; doi:10.1002/advs.202102104)
Supplement: Supplementary file 1 — Supporting Information [file ADVS-8-2102104-s001.pdf]

WILEY-VCH

© 2021 Wiley-VCH GmbH

ADVANCED SCIENCE Open Access

Supporting Information

for *Adv. Sci.*, DOI: 10.1002/advs.202102104

High-Speed and Continuous-Wave Programmable Luminescent Tags Based on Exclusive Room Temperature Phosphorescence (RTP)

*Max Gmelch, Tim Achenbach, Ausra Tomkeviciene, and Sebastian Reineke**

Supporting Information

High-Speed and Continuous-Wave Programmable Luminescent Tags Based on Exclusive Room Temperature Phosphorescence (RTP)

*Max Gmelch, Tim Achenbach, Ausra Tomkeviciene, and Sebastian Reineke**

Derivation of the average oxygen quenching rate k_{O2}

$$\tau_{\mathrm{O2}} = \frac{1}{k_{\mathrm{r}} + k_{\mathrm{nr}} + k_{\mathrm{O2}}} \tag{S1}$$

$$\tau_{\mathrm{N2}} = \frac{1}{k_{\mathrm{r}} + k_{\mathrm{nr}}} \tag{S2}$$

$$\frac{1}{\tau_{\mathrm{O2}}} - \frac{1}{\tau_{\mathrm{N2}}} = k_{\mathrm{r}} + k_{\mathrm{nr}} + k_{\mathrm{O2}} - k_{\mathrm{r}} + k_{\mathrm{nr}} = k_{\mathrm{O2}} \tag{S3}$$

While τ_{O2} is the phosphorescence lifetime in the presence of oxygen, τ_{N2} is the phosphorescence lifetime in the absence of oxygen, k_{r} is the radiative and k_{nr} the intrinsic nonradiative rate.

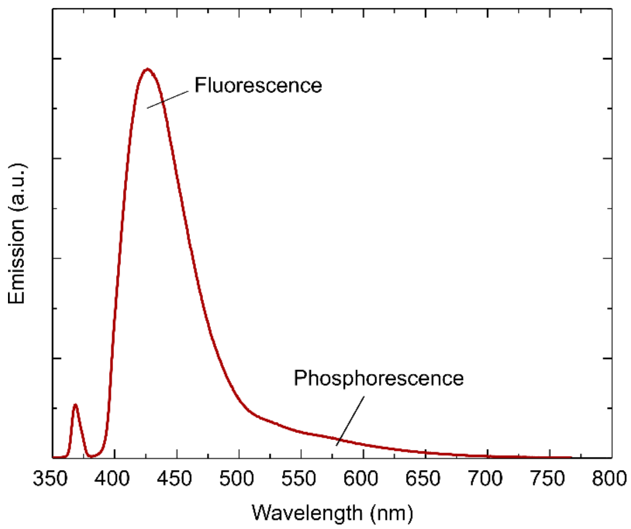

Figure S1. Emission of a PMMA:NPB (5 wt%) sample in nitrogen atmosphere. The weak phosphorescence at 550 nm is outcompeted by the intense fluorescence.

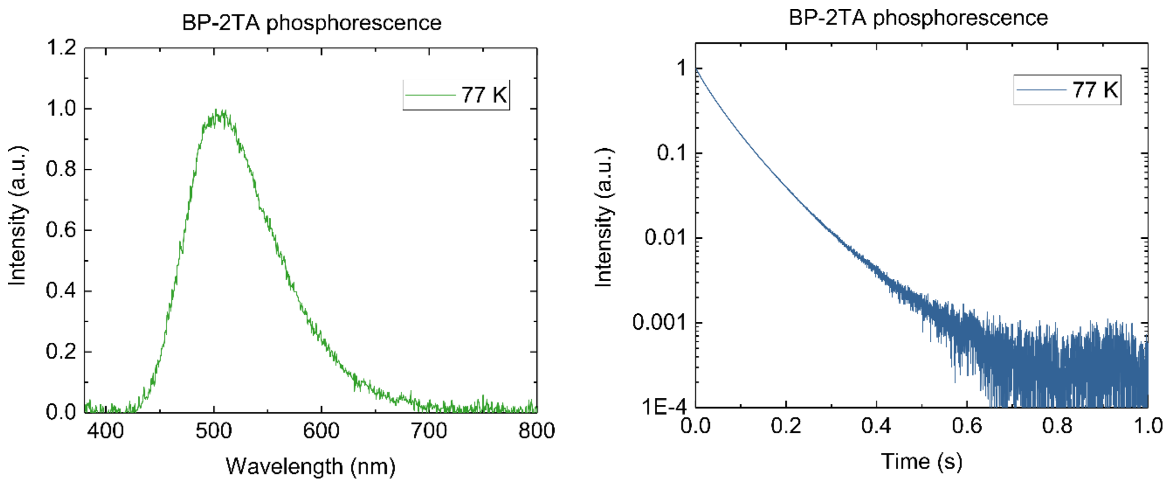

Figure S2. Phosphorescence spectrum and decay of a PMMA:BP-2TA (5 wt%) sample measured at a temperature of 77 K in liquid nitrogen shortly after excitation turnoff.

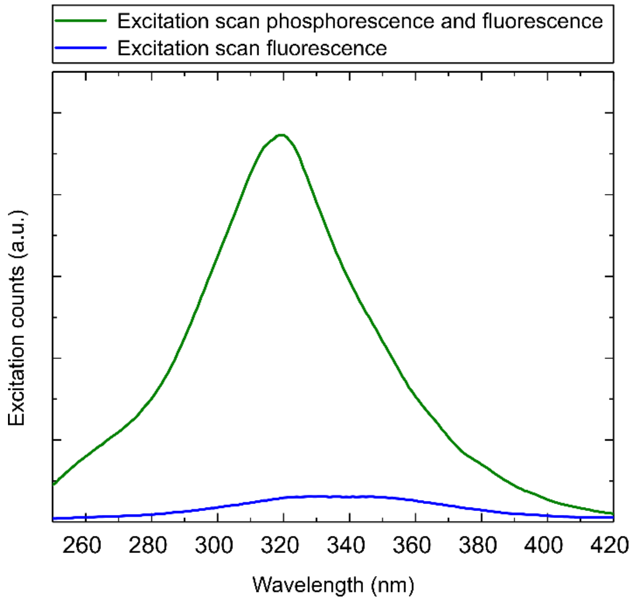

Figure S3. Excitation scans of PMMA:BP-2TA (5 wt%) The fluorescence excitation scan (blue) was recorded using a PLT sample before activating the phosphorescence, the combined phosphorescence and fluorescence scan (green) after activation of phosphorescence. For both measurements, the emission wavelength was set to 470 nm.

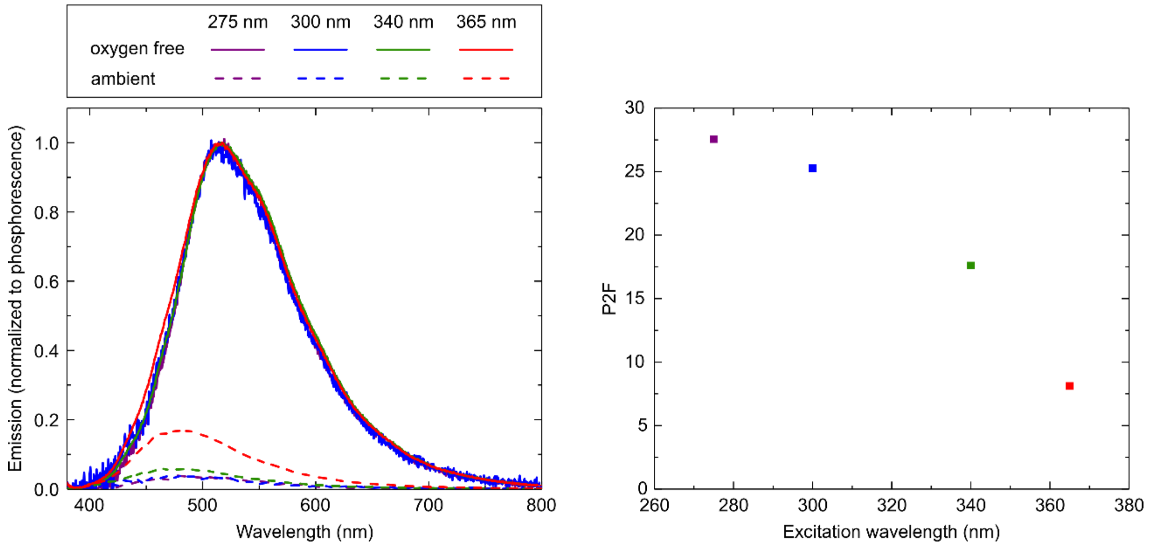

Figure S4. Excitation wavelength dependence of PMMA:BP-2TA (5 wt%). Spectra were taken in ambient and nitrogen atmosphere to measure fluorescence and phosphorescence. To calculate the P2F ratio, the spectra were integrated from 400 nm to 750 nm.

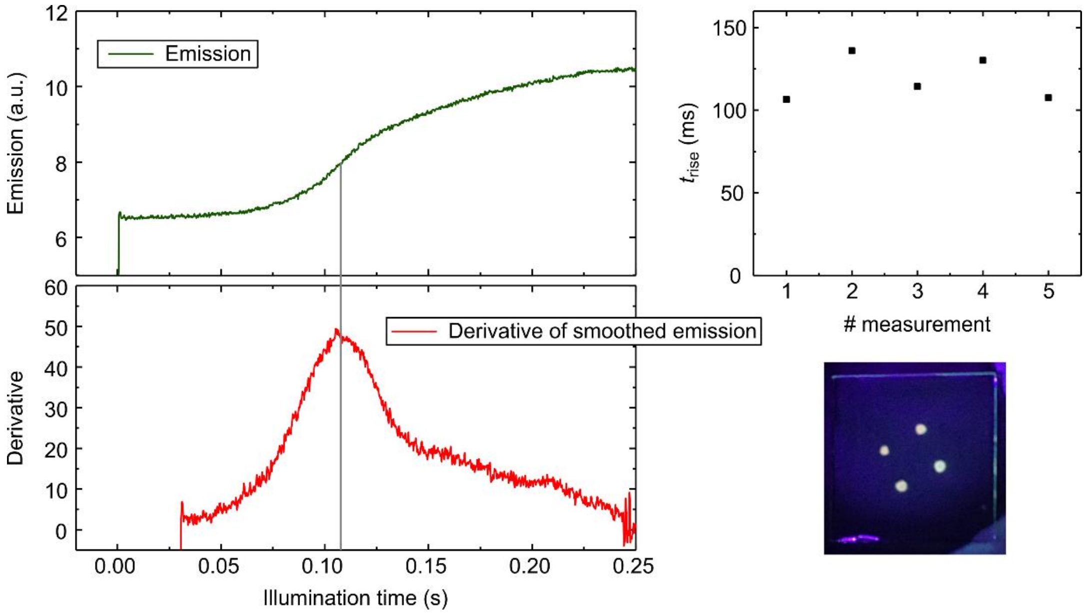

Figure S5. High-intensity UV activation of a cw-PLT containing PMMA:BP-2TA (5 wt%). Using a high-intensity UV source, a PLT was illuminated through a 1x1 mm² pinhole and the emission increase was monitored. Right behind the pinhole, the UV intensity was 2.3 W cm^{-2}. Due to scattered light, the sample got additionally partly illuminated in the area around the pinhole with lower intensity. Therefore, the measured rise of the emission did not stop sharply after activation of the area right behind the pinhole, leading to a smeared out plateau of the emission. Thus, for extracting the rise time, here the inflection point of the emission rise was taken as t_{rise}. To do so, the maximum of the first derivative was used. Five measurements were acquired, leading to a mean value of t_{rise} = 120 ± 20 ms. The photograph shows four different measurements. The different spot sizes result from the activation of phosphorescence beyond the pinhole, leading to the smeared out plateau.

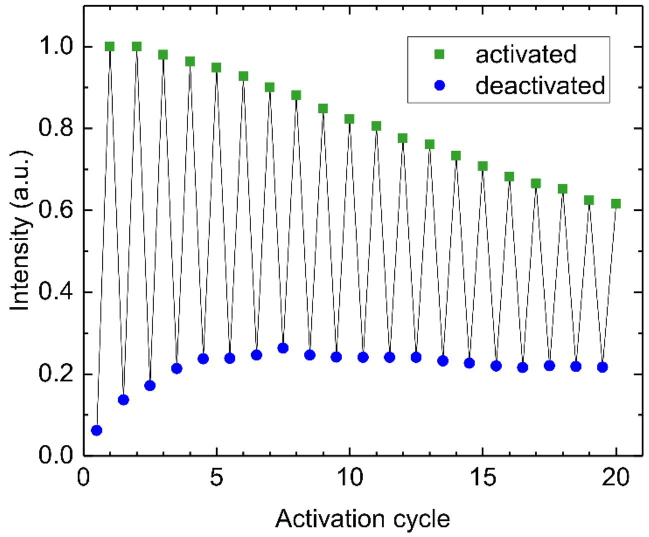

Figure S6. Result of 20 activation-deactivation cycles of a PMMA:BP-2TA (5 wt%) sample covered with an oxygen barrier layer. The intensities obtained are integrated spectra from 400 to 800 nm at the beginning and end of each cycle. For activation, the sample was illuminated with a high-intensity UV source. For deactivation, the sample was heated on a hotplate at 90°C for one minute, followed by a two-minute cooling period before the next activation. As cycling progresses, the sample slowly degrades and additional fluorescent emission emerges, reducing the P2F ratio and thus decreasing the performance of the tag. However, with a P2F ratio of ~2 after 20 cycles, the performance is still far above all the other emitters tested.

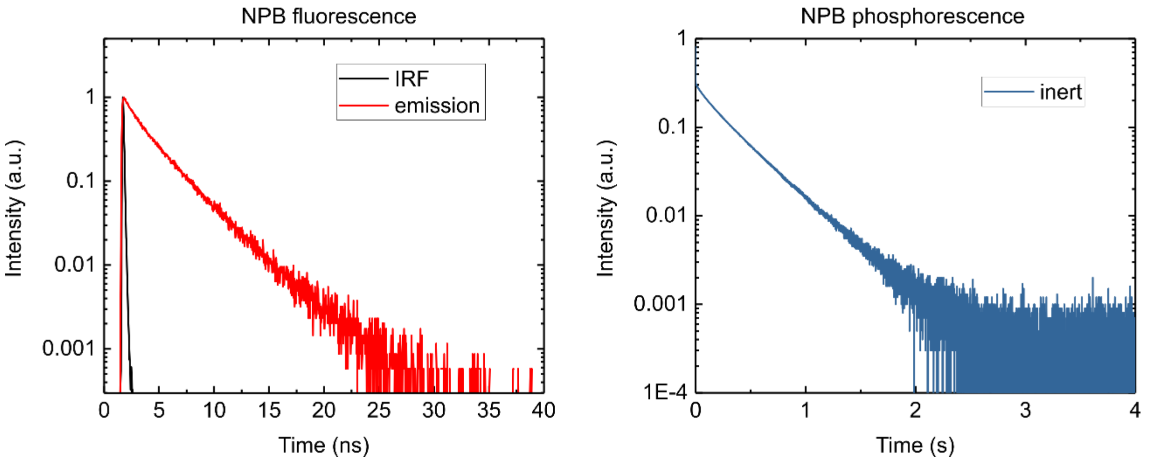

Figure S7. Fluorescence and phosphorescence decays of a PMMA:NPB (5 wt%) sample. The fluorescence decay was measured in ambient atmosphere; the phosphorescence decay was measured in nitrogen atmosphere.

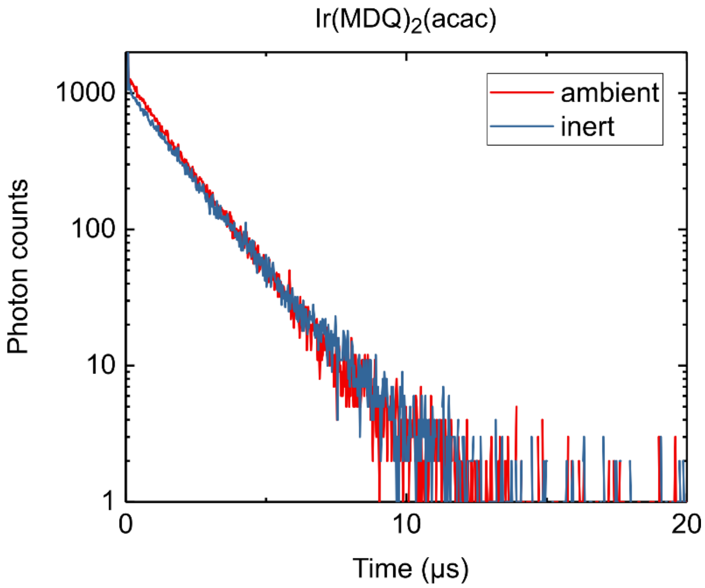

Figure S8. Phosphorescence decays of a PMMA:Ir(MDQ)$_2$(acac) (6 wt%) sample. The decays were measured in ambient and in inert nitrogen atmosphere.

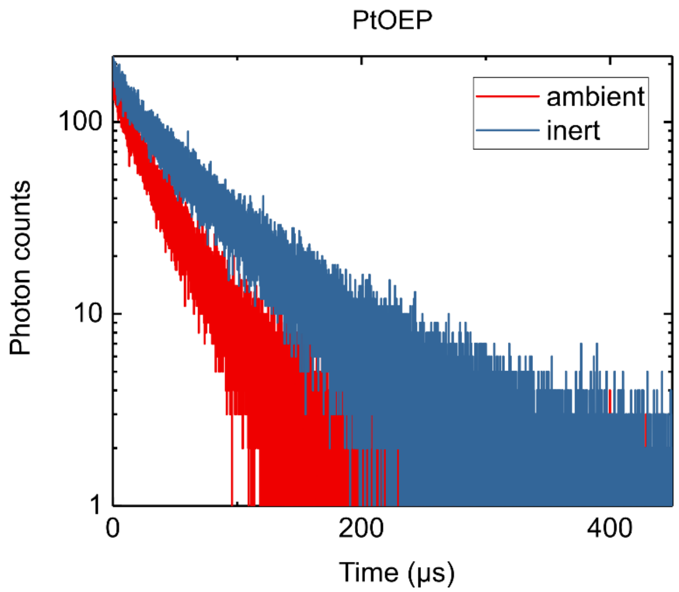

Figure S9. Phosphorescence decays of a PMMA:PtOEP (6 wt%) sample. The decays were measured in ambient and in inert nitrogen atmosphere.

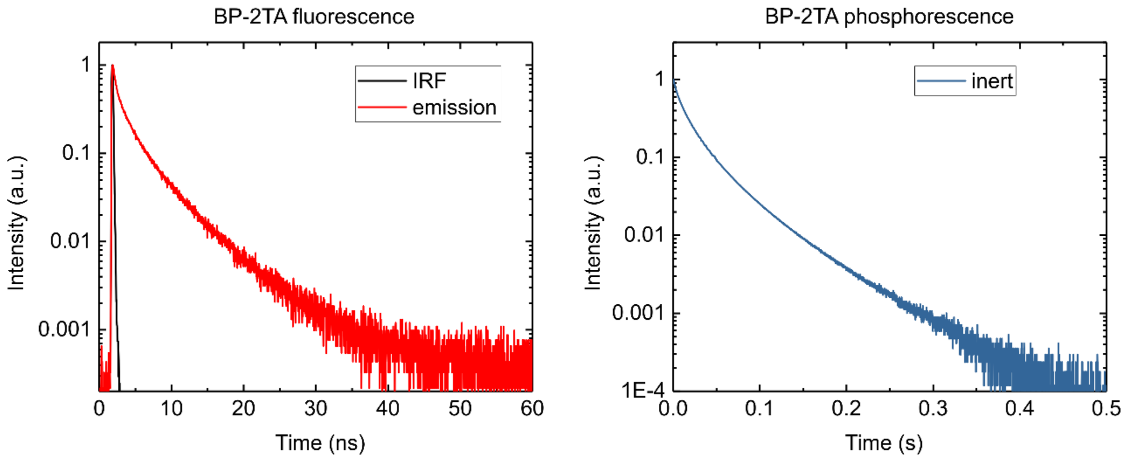

Figure S10. Fluorescence and phosphorescence decays of a PMMA:BP-2TA (5 wt%) sample. The fluorescence decay was measured in ambient atmosphere; the phosphorescence decay was measured in nitrogen atmosphere.
